# Versisterol, a new endophytic steroid with 3CL protease inhibitory activity from *Avicennia marina* (Forssk.) Vierh.†

**DOI:** 10.1039/d2ra00877g

**Published:** 2022-04-26

**Authors:** Marwa Elsbaey, Mahmoud A. A. Ibrahim, Mohamed-Elamir F. Hegazy

**Affiliations:** Pharmacognosy Department, Faculty of Pharmacy, Mansoura University Mansoura 35516 Egypt marwaelsebay1611@mans.edu.eg; Computational Chemistry Laboratory, Chemistry Department, Faculty of Science, Minia University Minia 61519 Egypt; Chemistry of Medicinal Plants Department, National Research Centre Giza 12622 Egypt

## Abstract

A new epoxy ergostane sterol, named versisterol, was isolated from *Aspergillus versicolor*, an endophytic fungus from *Avicennia marina*. The structure of the isolated compound was deduced by means of one- and two-dimensional NMR and high-resolution mass spectrometry. The absolute stereochemistry was elucidated by NOESY analysis, and experimental and calculated time-dependent density functional theory (TD-DFT) circular dichroism spectroscopy. Versisterol inhibited 3CL protease (3CL^pro^) with an IC_50_ value of 2.168 ± 0.09 μM. Binding affinities and molecular interactions of versisterol towards 3CL^pro^ were scrutinized and compared to lopinavir with the help of the combination of docking computations and molecular dynamics (MD) simulation. *In silico* calculations demonstrated a comparable binding affinity of versisterol with a docking score of −9.4 kcal mol^−1^, and MM-GBSA binding energy over 200 ns MD simulation of −29.1 kcal mol^−1^, with respect to lopinavir (−9.8 and −32.2 kcal mol^−1^, respectively). These findings suggested that versisterol can be an auspicious prototype for developing new 3CL^pro^ drug candidates against COVID-19.

## Abstract 2

A new epoxy ergostane sterol, named versisterol, was isolated from *Aspergillus versicolor*, an endophytic fungus from *Avicennia marina*.

## Graphical abstract

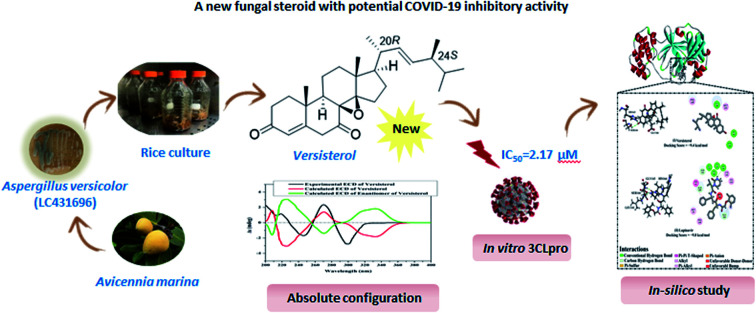

## 1. Introduction

Owing to their diverse biosynthetic capacity, endophytic fungi represent an enormous pool for novel secondary metabolites. A literature review shows that the genus *Aspergillus* is the most dominant, representing an untapped source of diverse bioactive compounds.^1^ By way of example and not limitation, steroids,^2^ bisabolenes,^3^ indoloditerpens,^4^ meroterpenes,^5^ quinazolines,^6^ xanthones and anthraquinones,^7^ cyclopeptides and polyketides^8^ were previously reported from *Aspergillus versicolor*.

*Avicennia marina* (Forssk.) Vierh. is a widespread mangrove in Egypt.^9^ Through our previous investigation on the endophytic diversity of *A. marina*, we have reported several secondary metabolites from *A. versicolor*,^5,10^*A. amstelodami*^11^ and *Cladosporium cladosporioides*.^12^ In this manuscript, we report a new epoxy steroid from the rice culture of *A. versicolor*, an endophytic fungus isolated from *A. marina* fruits. In late 2019, the pandemic outbreak of coronavirus (COVID-19) appeared. A novel severe acute respiratory syndrome coronavirus (SARS-CoV-2) caused the pandemic.^13^ To date, there is no effective treatment; COVID-19 has not been only a major health problem, it is causing the worst economic crisis as well. The proteolytic processing of SARS-CoV-2 is mediated by 3-chymotrypsin-like protease (3CL^pro^), also named the main protease (M^pro^). The 3CL^pro^ has a main role in viral replication; therefore it is a prospective target for anticoronaviruses screening.^13^ In this work, the 3CL^pro^ inhibitory activity of versisterol was investigated. Molecular interaction and binding affinity of versisterol with 3CL^pro^ were foretold and compared to lopinavir (an antiviral protease inhibitor) with the assistance of molecular docking technique. The docked versisterol–3CL^pro^ complex was thereafter subjected to molecular dynamics (MD) simulations throughout 200 ns. Besides, the corresponding binding affinity and steadiness were estimated.

## 2. Results and discussion

### 2.1. Identification of versisterol

**Fig. 1:**
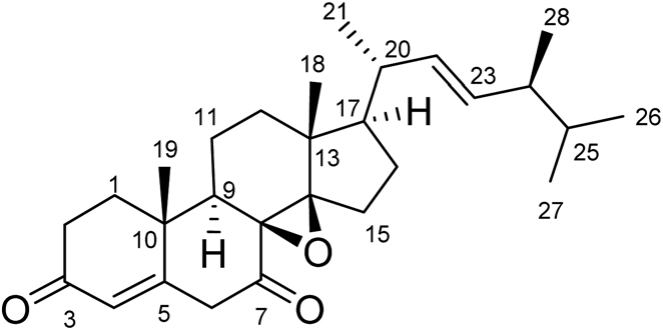
The structure of versisterol isolated from *A. vsersicolor*.

Versisterol (Fig. 1) was purified as a rose-colored amorphous material. It was obtained as a minor metabolite from the fermented rice culture of *A. versicolor*.

**Fig. 2:**
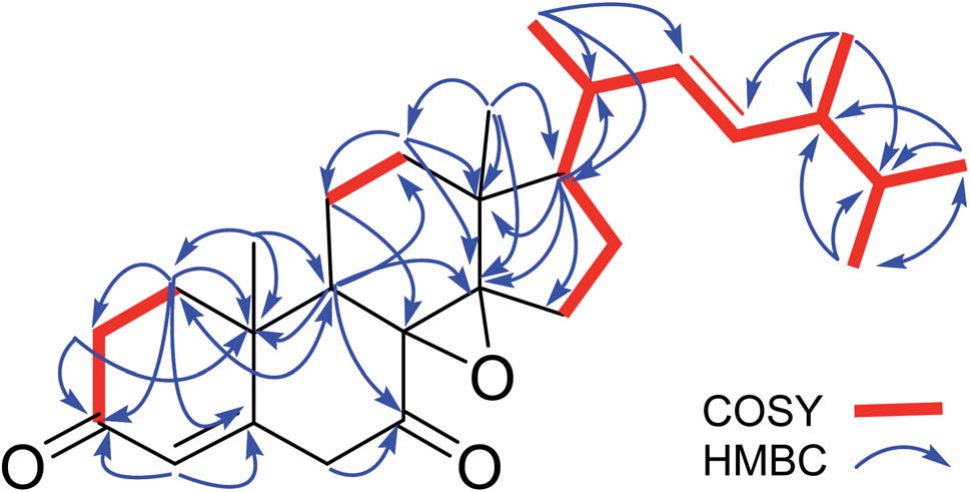
Key COSY and HMBC correlations for versisterol.

**Fig. 3:**
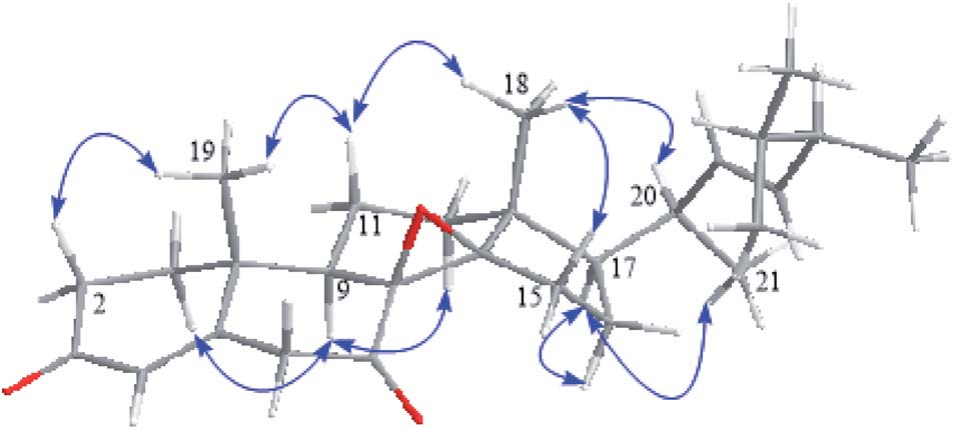
Key NOESY correlations for versisterol.

The molecular formula of versisterol was determined as C_28_H_40_O_3_ based on HR-ESIMS peak at *m*/*z* 447.2925 [M + Na]^+^ (calcd 447.2875). The previous molecular formula suggested nine degrees of saturation. Interpretation of 1 and 2D NMR spectral data (Table 1, Fig. 2 and 3) established the presence of a steroidal nucleus with 6 methyls, 7 methylenes, 8 methines and 7 quaternary carbons. The data also showed the presence of four olefinic carbons at *δ*_C_ 159.3, 126.5, 136.0, and 134.4; in addition to two carbonyl carbons at *δ*_C_ 201.7, and 216.8.

**Table 1:** NMR spectroscopic data of compound 1 (CD_3_OD, 600 and 150 MHz)

H/C	*δ*_C_ (m)	*δ*_H_ (m, *J* in Hz)
1	40.0 (CH_2_)	1β: 2.02 (m)
		1α: 2.07 (m)
2	39.0 (CH_2_)	2β: 2.64 (m)
		2α: 2.39 (m)
3	201.7 (c)	—
4	126.5 (CH)	6.16 (br d)
5	159.3 (c)	—
6	41.4 (CH_2_)	6β: 2.57 (d, 10.8)
		6α: 2.63 (d, 10.8)
7	216.8 (c)	—
8	66.9 (c)	—
9	51.0 (CH)	2.86 (dd, 14.1, 5.5)
10	37.4 (C)	—
11	25.9 (CH_2_)	11β: 1.98 (m)
		11α: 2.12 (m)
12	38.5 (CH_2_)	12β: 1.91 (m)
		12α: 1.72 (m)
13	55.7 (c)	—
14	64.1 (c)	—
15	35.2 (CH_2_)	15β: 2.58 (m)
		15α: 2.38 (m)
16	24.7 (CH_2_)	16β: 1.71 (m)
		16α: 1.96 (m)
17	49.7^a^ (CH)	1.70 (m)
18	17.4 (CH_3_)	0.99 (s)
19	24.2 (CH_3_)	1.29 (s)
20	38.6 (CH)	2.49 (m)
21	24.1 (CH3)	1.12 (d, 7.1)
22	134.3 (CH)	5.33 (m)
23	136.0 (CH)	5.33 (m)
24	44.7 (CH)	1.88 (m)
25	34.4 (CH)	1.48 (m)
26	20.1 (CH_3_)	0.84 (d, 6.8)
27	20.5 (CH_3_)	0.86 (d, 6.8)
28	18.1 (CH_3_)	0.94(d, 6.8)

a Masked by solvent signal.

The presence of four methyls showing the doublet splitting pattern at *δ*_H_ 1.12 (CH_3_-21), 0.84 (CH_3_-26), 0.86 (CH_3_-27) and 0.94 (CH_3_-28), in addition to the olefinic carbons at *δ*_C_ 134.3 (C-22) and 136.0 (C-23) are a typical feature for the unsaturated alkyl side chain of ergosterol.^14^ This was established from the HMBC correlations of H_3_-21 to C-17, C-20, and C-22; H_3_-28 to C-23 and C-25; as well as H_3_-26 and H_3_-27 to C-24 and C-25. The methines at *δ*_H_/C 2.86/51.0; 1.70/49.7; 2.49/38.6 were assigned to C-9, C-17, and C-20, respectively. This was established from the HMBC correlations of H_3_-19 to C-9; H_3_-18 and H_3_-21 to C-17; H_3_-21 and H-22 to C-20.

An α–β unsaturated carbonyl fragment was established from the shielded olefinic methine at *δ*_C_ 126.5 (C-4), deshielded olefinic quaternary carbon at *δ*_C_ 159.3 (C-5) and the carbonyl group at *δ*_C_ 201.7 (C-3). This was established from the HMBC correlations of H-4 to C-3 and C-5. In addition, another carbonyl group was assigned at position 7, resonating at *δ*_C_ 216.8 (C-7); this was established from the HMBC correlations of H-9 to C-7; and H-6 to C-7.

The two oxygenated quaternary carbons at *δ*_C_ 66.9 (C-8) and 64.1 (C-14) were assigned to an epoxy moiety at positions 8 and 14. This was confirmed from HMBC correlations of H-9, H_3_-18, H-12, and H-15 to C-14; and H-11/C-8.

The relative configuration was determined from the detailed analysis of the NOESY correlations (Fig. 3). The correlations of H_3_-19 with H-2β and 11β; H-11β with H_3_-18; H_3_-18 with H-15β; and H-15β with H-16β revealed that these protons are β-positioned. Meanwhile, the correlations of H-9 with H-1α, H-6α and H-12α; and H-17 with H-16α suggested that these protons are α-positioned. The correlations of H_3_-18 with H-20 as well as H_3_-21 with H-17 suggested (20*R*) configuration.^15^ Furthermore, regarding biogenesis, the 20*S*-epimer of ergosterol probably does not exist biologically. This theoretical restriction does not apply at C-24, and both C-24 epimers could naturally exist, although apparently not in fungi.^16^

In general, it is reported that fungi and algae produce sterols with the 24β configuration, whereas those produced in most vascular plants possess the 24α configuration.^17^ Furthermore, the chemical shifts of the side-chain carbons can permit the determination of the absolute configuration at C-24 in sterols. Referring to the C-24 epimers of 24-methylchlolesta-5,22-diene-3β-ol, the resonance of CH_3_-28 in versisterol (*δ*_C_ 18.10) was constituent with the 24β-epimer (*δ*_C_ 18.08), not with the 24α-epimer, in which CH_3_-28 is reported to specifically resonate at (*δ*_C_ 17.6 ± 0.1).^17^ Based on this evidence, the (24*S*) configuration was concluded.

The next step was to determine the configuration of the epoxy group. According to the literature, the oxirane ring is almost perpendicular to the molecular plane,^15^ and it was placed in the β-orientation based on the CD calculations. The absolute configuration of the compound was determined by the time-dependent density functional theory (TD-DFT) ECD calculations. To further confirm the β-orientation of the epoxy group, the chemical shift of C-8 and C-14 in versisterol were compared to the closely related (22*E*)-ergosta-22-ene-8,14-epoxy-3,7-dione (ehrenasterol).^18^ In versisterol, C-8 and C-14 resonated at *δ*_C_ 66.9 and 64.1, respectively; meanwhile, the α-oriented epoxy group in ehrenasterol resonated at *δ*_C_ 60.0 and 65.6, respectively. Furthermore, the deshielding of CH_3_-18 in versisterol (*δ*_C_ 17.4, *δ*_H_ 0.99) as compared to ehrenasterol (*δ*_C_ 15.3, *δ*_H_ 0.75) may suggest that CH_3_-18 and the epoxy group are oriented in the same side *i.e.* the β-orientation.^19^

**Fig. 4:**
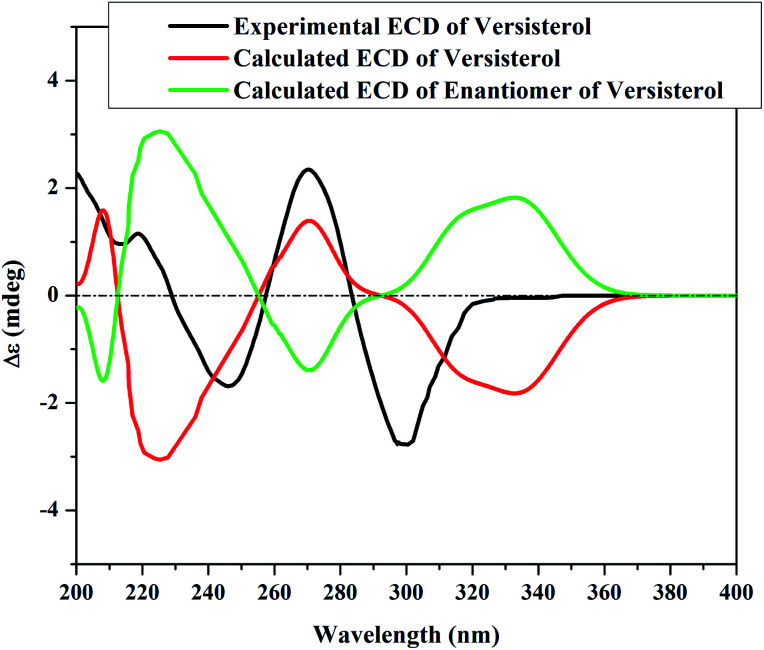
Experimental and TD-DFT-simulated electronic circular dichroism (ECD) in methanol for versisterol.

The experimental ECD spectrum (Fig. 4) was in good agreement with the theoretical ECD of (22*E*,20*R*)-8β,14β-epoxy-ergosta-4,22-diene-3,7-dione.

Accordingly, the compound was elucidated as (22*E*,20*R*,24*S*)-8β,14β-epoxy-ergosta-4,22-diene-3,7-dione, and trivially named versisterol. The compound is a new derivative of ergosterol, which is the most abundant sterol in fungal cell membranes.^20^

### 2.2. 3CL^pro^ inhibitory activity

To our current date, there is no effective treatment of COVID-19. Lopinavir and ritonavir are protease inhibitors that are commonly used for the treatment of HIV infection. The combination has been repurposed for COVID-19; however, it is still going under clinical trials.^21^

Natural products have always been exploited in the pursuit of new drugs. The 3CL^pro^ enzyme is a prospective target for anticoronaviruses drugs.^13^ Versisterol, a new ergosterol derivative isolated from the endophytic *A. versicolor*, in this study, was tested for its 3CL^pro^ inhibition activity (Table 2). It showed an IC_50_ value of 2.168 ± 0.09 μM, which is about fifteen times of lopinavir (IC_50_ value of 0.148 ± 0.01 μM). It is worth noting that several steroids are reported to inhibit bovine chymotrypsin.^22^ In addition, ergosterol peroxide is reported to demonstrate antiviral and immunomodulatory activity against porcine delta coronavirus.^23^ The results, in addition to previous literature, suggest that versisterol is a promising skeleton for designing new drugs against COVID-19. These results prompted us to investigate the binding affinities and molecular interactions of versisterol towards 3CL^pro^ and compare it to lopinavir employing molecular docking and molecular dynamics (MD) simulations.

**Table 2:** The 3CL protease inhibitory activity^a^

Compound	IC_50_ (μM)
Versisterol	2.168 ± 0.09
Lopinavir	0.148 ± 0.01

a The data are expressed as mean ± standard deviation.

### 2.3. Molecular docking

**Fig. 5:**
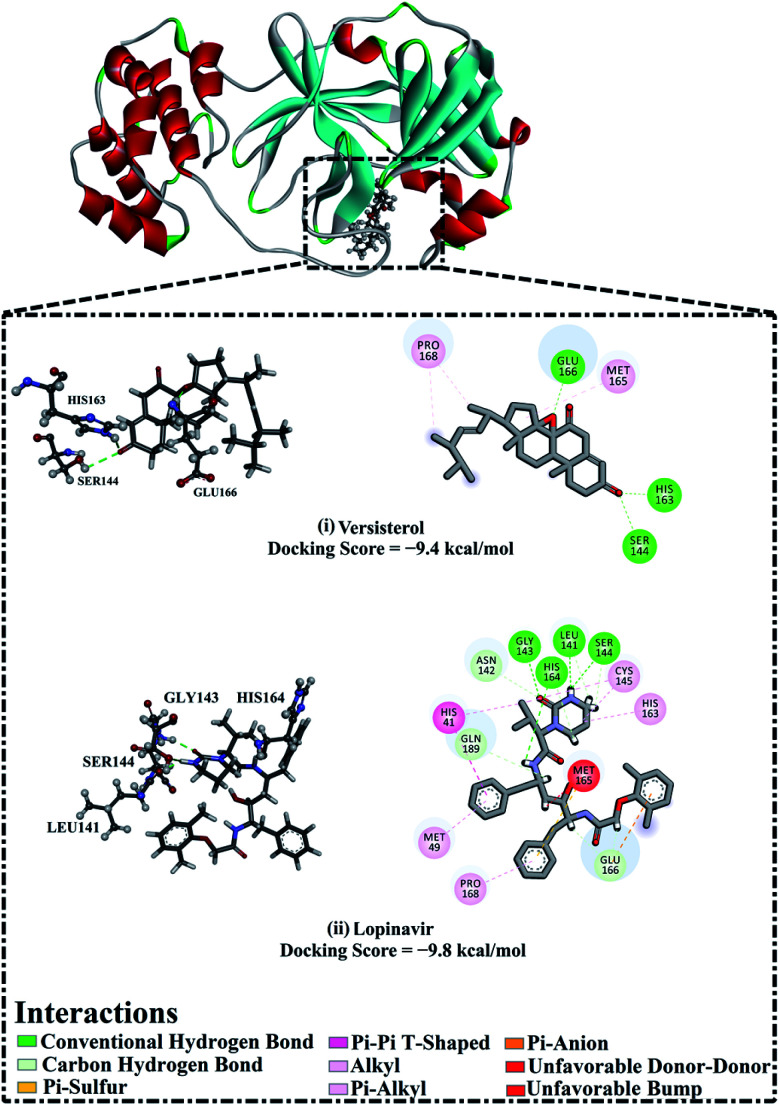
3D and 2D representations as well as the predicted docking scores of (i) versisterol and (ii) lopinavir with 3-chymotrypsin-like protease (3CL^pro^).

AutoDock4.2.6 software was used to anticipate the binding affinities and molecular features of the isolated versisterol and lopinavir complexed with 3CL^pro^. The portended binding affinities, 3D in addition to 2D visualizations of the docking poses of the investigated inhibitors within the binding pocket of 3CL^pro^ are shown in Fig. 5. From the data in Fig. 5, versisterol elucidated an auspicious binding affinity towards 3CL^pro^ with a docking score of −9.4 kcal mol^−1^. The prodigious potentiality of versisterol as a 3CL^pro^ inhibitor is ascribed to its capacity to exhibit various hydrogen bonds, hydrophobic, in addition to van der Waals interactions with the most important amino acids within the binding pocket (Fig. 5). Precisely, the oxygen atom of the oxirane ring forms a hydrogen bond with the backbone NH of GLU166 with a bond length of 1.71 Å (Fig. 5). Besides, the CO group of cyclohexanone exhibits two hydrogen bonds with the hydroxy group of SER144 and the imidazole of HIS163 with bond lengths of 2.72 and 1.96 Å (Fig. 5). In comparison with versisterol, lopinavir demonstrated an almost identical binding affinity against 3CL^pro^ with a docking score of −9.8 kcal mol^−1^, exhibiting four hydrogen bonds with LEU141, SER144, HIS163, as well as GLY143 with bond lengths in the range of 1.96 to 3.09 Å (Fig. 5).

### 2.4. Molecular dynamics simulations

**Fig. 6:**
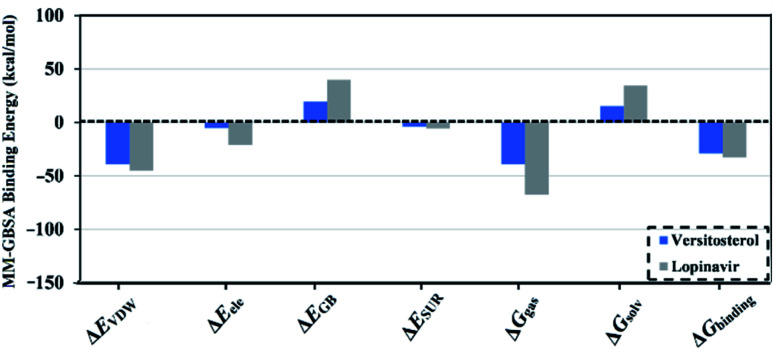
Decomposition of MM-GBSA binding energies for the inspected inhibitors complexed with 3-chymotrypsin-like protease (3CL^pro^) throughout 200 ns MD simulations.

Molecular dynamics (MD) simulations inspect the steadiness of the inhibitor–target complexes, structural specifics, orientational flexibilities, in addition to the thoroughness of inhibitor–target binding affinities.^24,25^ As a consequence, the investigated inhibitors complexed with 3CL^pro^ were submitted to MD simulations pursued by binding free energy estimations using the molecular mechanics-generalized born surface area (MM-GBSA) approach. The computed MM-GBSA binding energies according to the gathered trajectories over 200 ns are represented in Fig. 6. As shown in Fig. 6, versisterol revealed a favorable binding affinity with an average Δ*G*_binding_ of −29.1 kcal mol^−1^ compared to lopinavir (calc. −32.2 kcal mol^−1^). The current results were in line with the experimental data, manifesting the comparable binding affinity of versisterol as a 3CL^pro^ inhibitor.

To explore the most significant interactions between the inhibitor and enzyme, MM-GBSA binding free energies of the scrutinized inhibitors complexed with 3CL^pro^ were decomposed and depicted in Fig. 6. As shown in Fig. 6, it is evident that the binding affinities of versisterol and lopinavir were predominated *via E*_vdw_ interactions with average values of −39.1 and −45.2 kcal mol^−1^, respectively. *E*_ele_ interactions were convenient with average values of −5.4 and −21.1 kcal mol^−1^ for versisterol and lopinavir, respectively (Fig. 6). Together these findings supply quantitative data of the binding free energies of versisterol and lopinavir as anti-COVID-19 drug candidates.

### 2.5. Post-MD analyses

In order to otherwise establish the constancy and behavior of versisterol in complex with 3CL^pro^, structural and energetical analyses were accomplished throughout the simulation time of 200 ns and compared to those of lopinavir. Monitoring of the conformational immutability of the investigated complex was fulfilled *via* inspecting root-mean-square deviation (RMSD) and binding energy per-frame.

#### 2.5.1. Binding energy per-frame

**Fig. 7:**
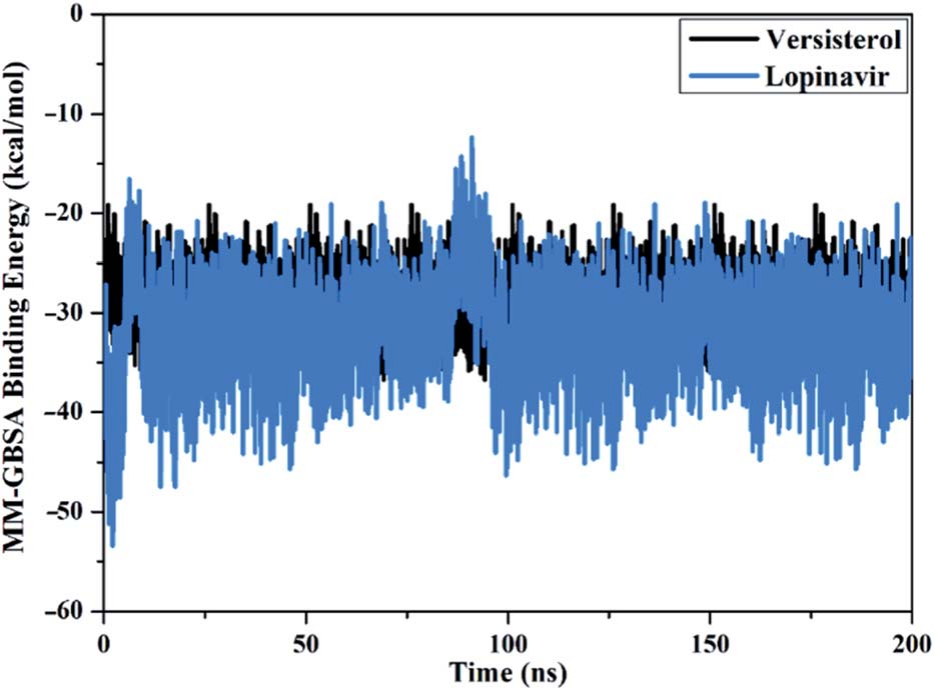
Estimated MM-GBSA binding energy per-frame for versisterol (in black), and lopinavir (in light blue) with 3-chymotrypsin-like protease (3CL^pro^) throughout 200 ns MD simulations.

The thorough structural immutability of versisterol– and lopinavir–3CL^pro^ complexes was inspected for a period of 200 ns MD simulations *via* mensurating the correlation between the binding energy per-frame and time (Fig. 7). Global steadiness for versisterol and lopinavir demonstrated average binding affinities (Δ*G*_binding_) of −29.1 and −32.2 kcal mol^−1^, respectively (Fig. 7). Based on the binding energy per frame analysis, all inspected complexes maintained steadiness during the 200 ns MD simulations.

#### 2.5.2. Root-mean-square deviation

**Fig. 8:**
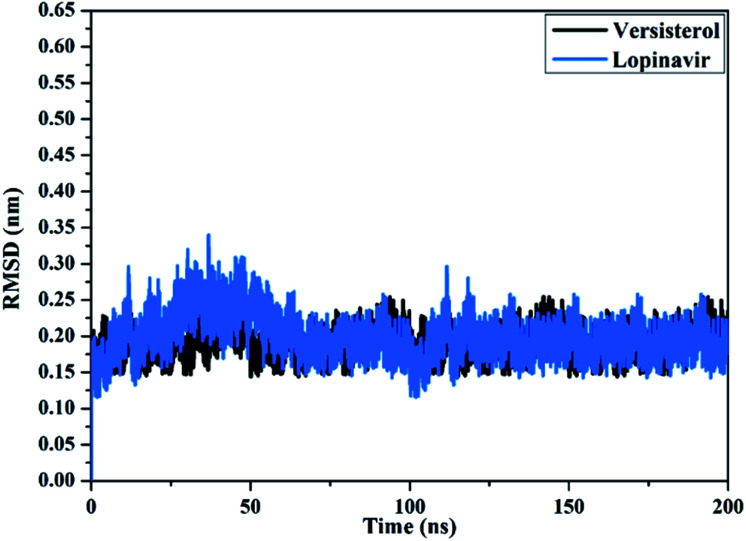
Root-mean-square deviation (RMSD) of the backbone atoms from the starting structure for versisterol (in black) and lopinavir (in light blue) with the 3-chymotrypsin-like protease (3CL^pro^) throughout 200 ns MD simulations.

To examine the conformational steadiness of the versisterol– and lopinavir–3CL^pro^ complexes, the root-mean-square deviation (RMSD) values of the backbone atoms of the entire system were evaluated (Fig. 8). Precisely, the evaluated RMSD values for the inspected complexes endured underneath 0.25 nm throughout the simulation time of 200 ns. The versisterol and lopinavir complexed with 3CL^pro^ attained the stationary state in the first 5 ns MD simulations and uninterrupted in a nearly stable state till the termination of the simulations. These findings asserted that the versisterol is tightly bonded and does not affect the comprehensive topology of 3CL^pro^.

## 3. Experimental

### 3.1. General experimental procedures

Nuclear magnetic resonance (NMR) measurements were conducted on Varian INOVA-600 (600 MHz and 150 MHz for ^1^H and ^13^C, respectively). Chemical shifts (*δ*) are expressed in ppm and coupling constants (*J*) are in Hz. A Bruker microTOF mass spectrometer was used for high-resolution mass measurement. ECD spectra were recorded at room temperature on a Jasco J815 spectrophotometer in 1 cm cuvettes. A PerkinElmer Model 343 polarimeter was used for recording the optical rotation. Column chromatography was performed using silica gel G 60-230 (Merck, Germany). The thin-layer chromatographic screening was made with Merck precoated silica gel F254 plates.

### 3.2. Fungal strain

The endophytic fungal strain was purified from the fruit of the mangrove *Avicennia marina* (Forssk.) Vierh. The mangrove was obtained from Kilo 17, Safaga, Red Sea, Egypt. It was identified as *Aspergillus versicolor* (GenBank accession no. LC431696). The detailed procedures for fungus isolation, purification and fermentation were described previously.^5^

### 3.3. Purification of versisterol

The fermentation media were extracted with EtOAc and defatted with *n*-hexane as previously described.^5^ The defatted ethyl acetate extract (9.7 g) was chromatographed through a silica gel column (28 × 4 cm, 164 g) using *n*-hexane/EtOAc as a solvent system. Fraction 3 (114.6 mg, eluted using 30% EtOAc in hexane gradient elution) was purified over a silica gel column (45 × 1.5, 120 g) using CH_2_Cl_2_/MeOH. Versisterol (1.6 mg) was eluted using 2% MeOH in CH_2_Cl_2_.

#### 3.3.1. Versisterol

Rose colored amorphous material; [α]^25^_D_ = +23.75 (c 0.01, MeOH); ^1^H and ^13^C NMR data (CD_3_OD) see Table 1; HR-ESIMS: *m*/*z* 447.2925 [M + Na]^+^ (calcd for C_28_H_40_O_3_Na, 447.2875).

### 3.4. ECD calculations

To predict the possible conformers for versisterol, conformational analysis was thoroughly performed within an energy window value set to 10 kcal mol^−1^ with the assistance of Omega2 software.^26^ Geometric optimization was first optimized for the resulted conformers at the B3LYP/6-31G*. Upon the optimized conformers, frequency calculations were then carried out to outline the nature of the local minima and evaluate the Gibbs free energies. The time-dependent density functional theory (TD-DFT) computations were then executed to calculate the first fifty excitation states by incorporating the polarizable continuum model (PCM) utilizing methanol as a solvent. The obtained electronic circular dichroism (ECD) spectra were Boltzmann-averaged, and SpecDis 1.71 was used to obtain ECD spectra.^27,28^ The Gaussian09 software was used to perform the quantum mechanical calculations.^29^

### 3.5. 3CL^pro^ inhibitory activity

Inhibition of 3CL^pro^ enzyme activity was measured using the Fluorogenic 3CL^pro^ Assay Kit (BPS Bioscience #79955, San Diego CA, USA) following the guidelines of the manufacturer's instructions.^30,31^

### 3.6. *In silico* drug discovery

#### 3.6.1. 3CL^pro^ preparation

The three-dimensional (3D) structure of the 3-chymotrypsin-like cysteine protease (3CL^pro^) enzyme of SARS-CoV-2 (PDB code 6LU7)^32^ was retrieved and employed as a template for all *in silico* computations. For the 3CL^pro^ preparation, all heteroatoms, crystallographic water molecules, in addition to ions, were removed from the 3CL^pro^ file for further study. Utilizing the H++ server, the protonation state of 3CL^pro^ was evaluated, and all missing hydrogens were inserted.^33^

#### 3.6.2. Inhibitor preparation

The chemical structures of versisterol and lopinavir were converted to the 3D structure using Omega2 software.^27,34^ Energy minimization was then performed utilizing an MMFF94S force field within SZYBKI software.^35^

#### 3.6.3. Molecular docking

All molecular docking computations were executed by AutoDock4.2.6 software.^36^ To undertake molecular docking estimations, the pdbqt file of 3CL^pro^ was prepared using the AutoDock protocol.^37^ The maximum number of energy evaluations (eval) was adjusted to 25 000 000. Additionally, the number of genetic algorithm (GA) run was set to 250. Other docking parameter options were preserved at their default settings. The grid map utilizing a grid box was prepared with the assistance of the AutoGrid program. A grid box with dimensions 60 Å × 60 Å × 60 Å positioned at the coordinates (*x*, *y*, *z*) of −13.069, 9.740, and 68.490 was utilized to encompass the entire active site of 3CL^pro^. The grid spacing value was adjusted to 0.375 Å. The partial atomic charges of the investigated compounds were determined using the Gasteiger method.^38^ The foreseen docking poses for every investigated inhibitor were processed utilizing a tight clustering analysis through the root-mean-square deviation (RMSD) tolerance of 1.0 Å. Additionally, the lowest energy orientation from the largest cluster was selected as a representative binding mode.

#### 3.6.4. Molecular dynamics and binding energy calculations

AMBER16 software was applied to conduct molecular dynamics (MD) simulations for the inspected compounds in complex with 3CL^pro^ for a period of 200 ns.^39^ The minutiae of the utilized MD simulations are explicated in ref. 40–44. In succinct, the investigated inhibitors were characterized with the aid of the General AMBER Force Field (GAFF2).^45^ AMBER force field 14SB was used to parameterize the 3CL^pro^.^46^ The restrained electrostatic potential (RESP) charge model was used to appoint the atomic partial charges of the investigated inhibitors at the HF/6-31G* level using Gaussian09 software.^29,47^ The docked 3CL^pro^–inhibitor complexes were centered at a cubic box with 15 Å × 15 Å × 15 Å and solvated using TIP3P water model. The investigated systems were neutralized *via* counterions and a salt concentration of 0.15 M NaCl was preserved. The solvated systems were submitted to energy minimization for 5000 steps. The minimized systems were gradually heated to 300 K throughout 50 ps. The annealed complexes were equilibrated for 1 ns. After the equilibration, the production MD run was carried out for 200 ns utilizing periodic boundary conditions and NPT ensemble. All MD simulations were conducted *via* the GPU of pmemd (pmemd.cuda) within the AMBER16 package on the CompChem GPU/CPU cluster (https://hpc.compchem.net). Molecular visualizations of the inhibitor–3CL^pro^ interactions were represented with the assistance of the Biovia Discovery studio software (Dassault Systemes of France).^48^

The binding affinities of the inspected inhibitors complexed with 3CL^pro^ were computed utilizing the molecular mechanical-generalized Born surface area (MM-GBSA) approach.^49^ For MM-GBSA computations, uncorrelated snapshots were recorded every 10 ps over the production stage. The MM-GBSA binding energy (Δ*G*_binding_) can be conceptually summarized as:Δ*G*_binding_ = *G*_complex_ − (*G*_compound_ + *G*_3CL^pro^_)

## 4. Conclusion

A new epoxy steroid, versisterol, was purified from the endophytic fungus, *A. versicolor*. The absolute configuration was fully established by analyzing experimental and calculated TD-DFT ECD. Versisterol inhibited SARS-CoV-2 3CL protease (3CL^pro^) with IC_50_ value 2.168 ± 0.09 μM. Molecular docking and molecular dynamics (MD) simulations were utilized to predict the binding affinities and modes of versisterol against 3CL^pro^ and compared to lopinavir (an antiviral protease inhibitor). Versisterol revealed good binding affinity against 3CL^pro^ with a docking score of −9.4 kcal mol^−1^ and Δ*G*_binding_ = −29.1 kcal mol^−1^ over 200 ns MD simulations. On the other hand, lopinavir exhibited a docking score of −9.8 kcal mol^−1^ and Δ*G*_binding_ = −32.2 kcal mol^−1^. Energetical and structural analyses over the 200 ns MD simulations unveiled the constancy of versisterol and lopinavir with 3CL^pro^. This study provides some clues about versisterol as a candidate inhibitor for SARS-CoV-2 3CL^pro^.

## Author contributions

Marwa Elsbaey: conceptualization, investigation, methodology, writing – review & editing; Mahmoud A. A. Ibrahim: formal analysis, software, writing – review & editing; Mohamed-Elamir Hegazy: conceptualization and supervision.

## Conflicts of interest

There are no conflicts to declare.

## Supplementary Material

RA-012-D2RA00877G-s001
