# Supplementary material for: Versisterol, a new endophytic steroid with 3CL protease inhibitory activity from Avicennia marina (Forssk.) Vierh
Source: RSC Adv. 2022 Apr 26;12(20):12583–9. doi: 10.1039/d2ra00877g (PMC9039987; doi:10.1039/d2ra00877g)
Supplement: RA-012-D2RA00877G-s001 [file RA-012-D2RA00877G-s001.pdf]

Electronic Supplementary Material (ESI) for RSC Advances.
This journal is © The Royal Society of Chemistry 2022

Versisterol, a new endophytic steroid with 3CL protease inhibitory activity

Marwa Elsbaey[a*], Mahmoud A. A. Ibrahim[b], Mohamed-Elamir F. Hegazy[c]

[a]Pharmacognosy Department, Faculty of Pharmacy, Mansoura University, Mansoura 35516, Egypt

[b]Computational Chemistry Laboratory, Chemistry Department, Faculty of Science, Minia University,Minia 61519, Egypt

[c]Chemistry of Medicinal Plants Department, National Research Centre, Giza 12622, Egypt

***Corresponding author:** Marwa Elsbaey, Department of Pharmacognosy, Faculty of Pharmacy, Mansoura University, 35516, Egypt
Email: marwaelsebay1611@mans.edu.eg
Tel.: 00201005437480; Fax: +02(050)2247496
Address: Department of Pharmacognosy, Faculty of Pharmacy, Mansoura University, Mansoura 35516, Egypt

Table of content

**Content**	**Page**
Figure S1: ^{1}H-NMR spectrum of versisterol (CD_3OD, 600 MHz).	4
Figure S2: ^{13}C-NMR spectrum of versisterol (CD_3OD, 150 MHz).	5
Figure S3: HSQC of versisterol.	6
Figure S4: HMBC of versisterol.	7
Figure S5: COSY of versisterol.	8
Figure S6: TCOSY of versisterol.	9
Figure S7: NOESY of versisterol.	10
Figure S8: Positive HRES-ESI mass spectrum of versisterol.	11

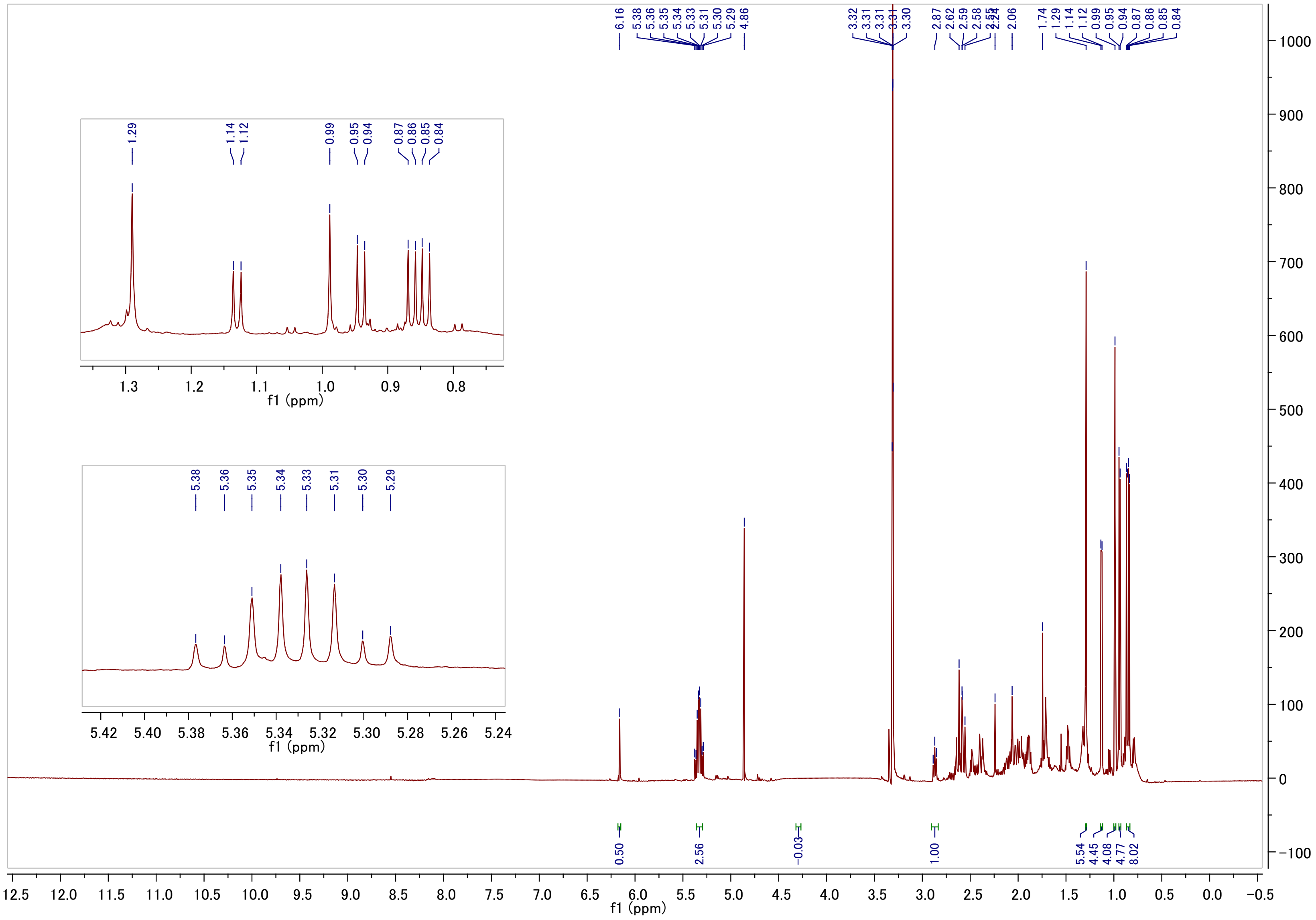

Figure S1: ^{1}H-NMR spectrum of versisterol (CD_3OD, 600 MHz).

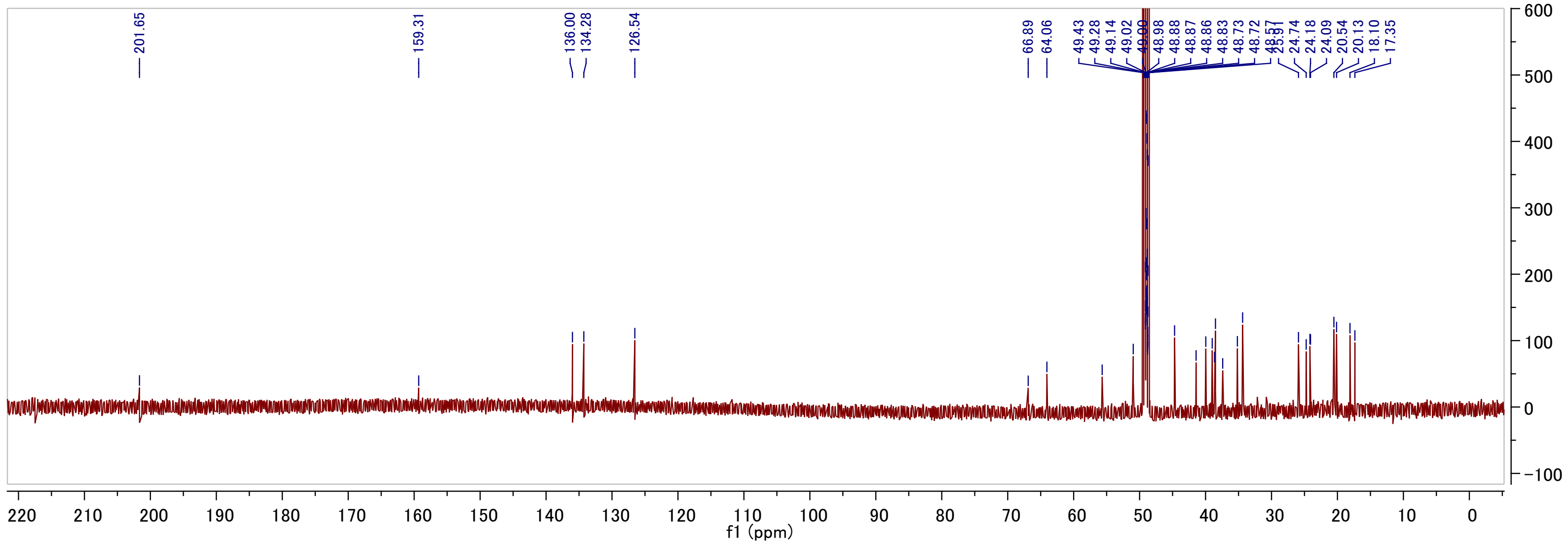

Figure S2: ^{13}C-NMR spectrum of versisterol (CD_3OD, 150 MHz).

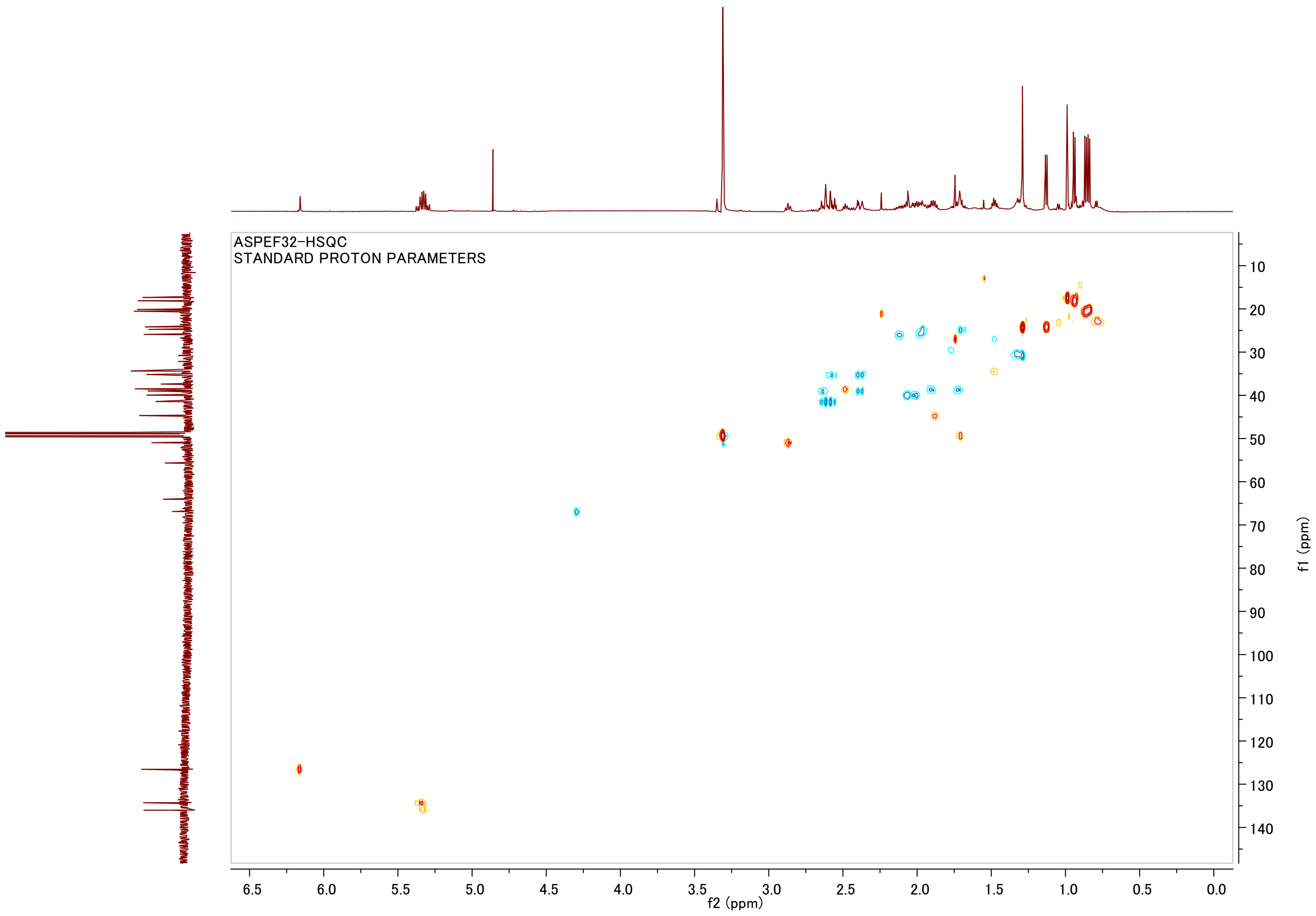

Figure S3: HSQC of versisterol.

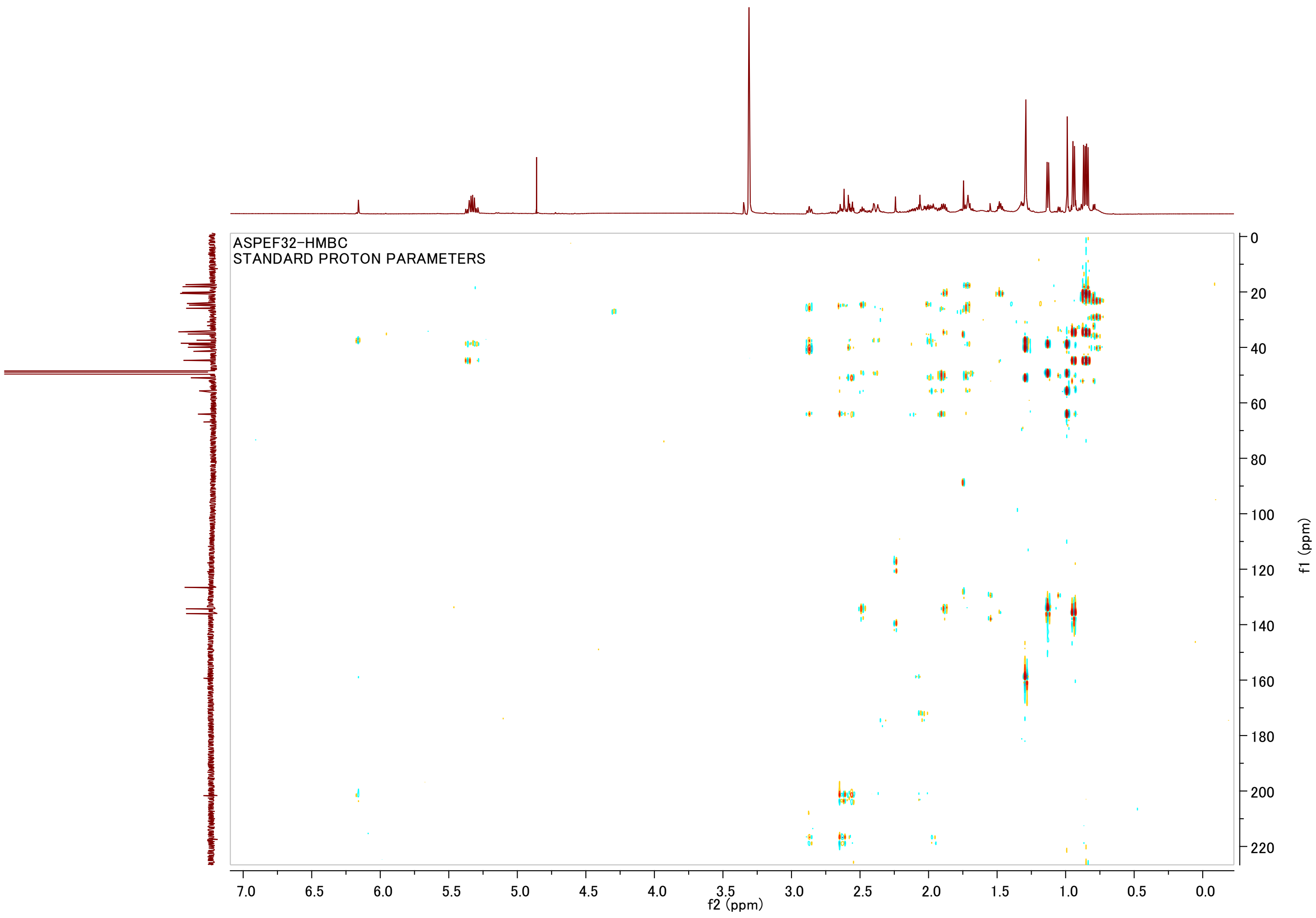

Figure S4: HMBC of versisterol.

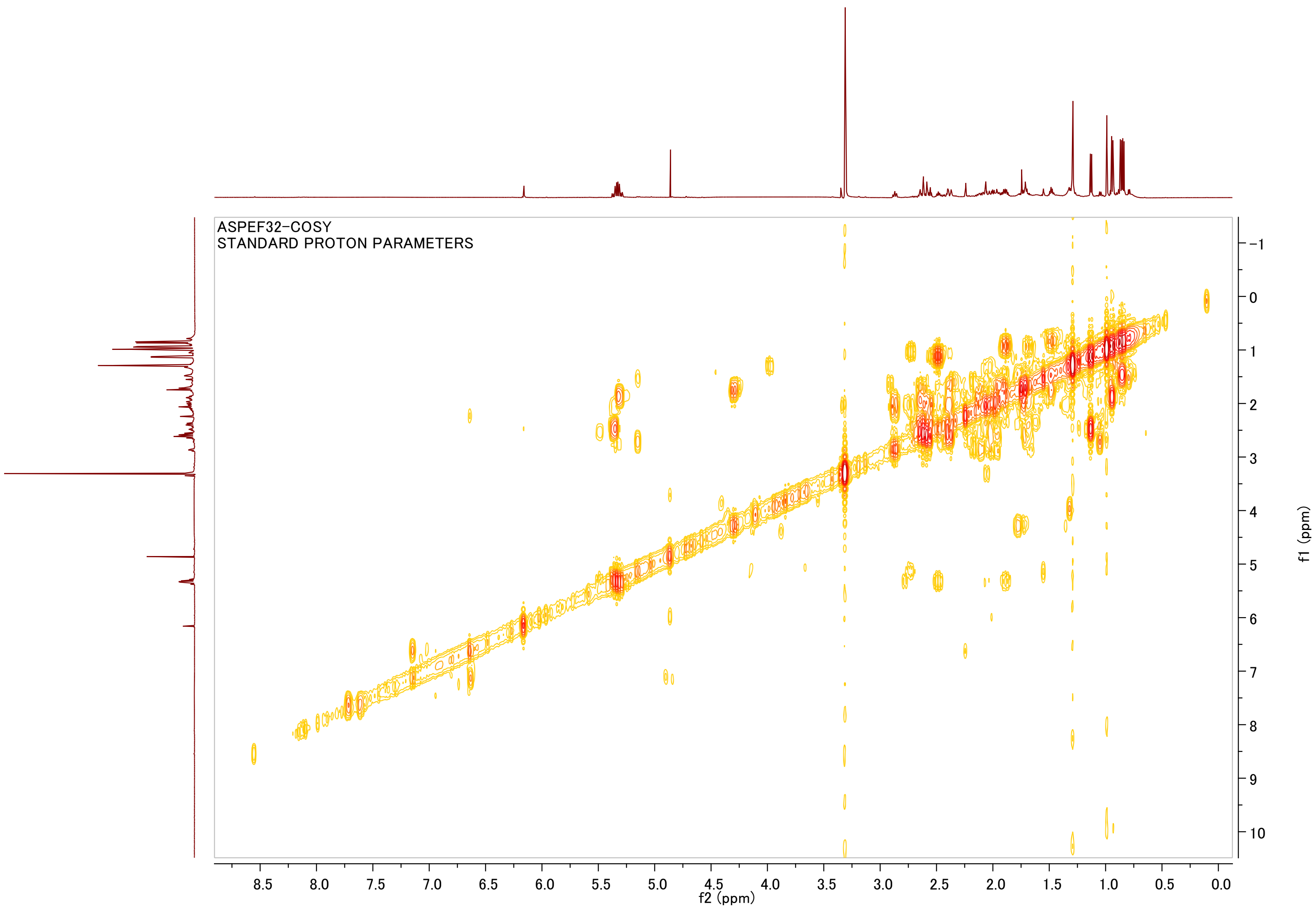

Figure S5: COSY of versisterol.

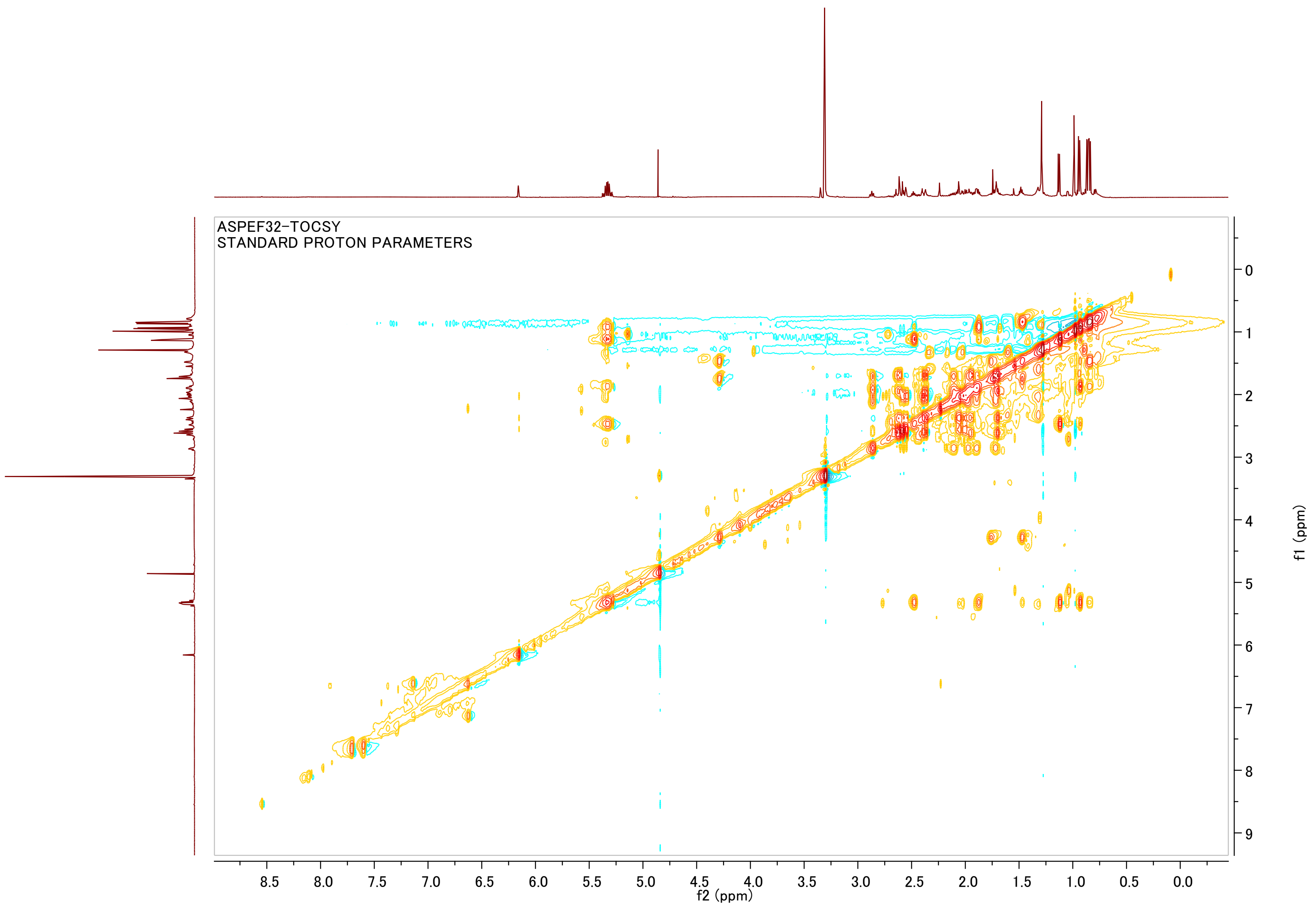

Figure S6: TCOSY of versisterol.

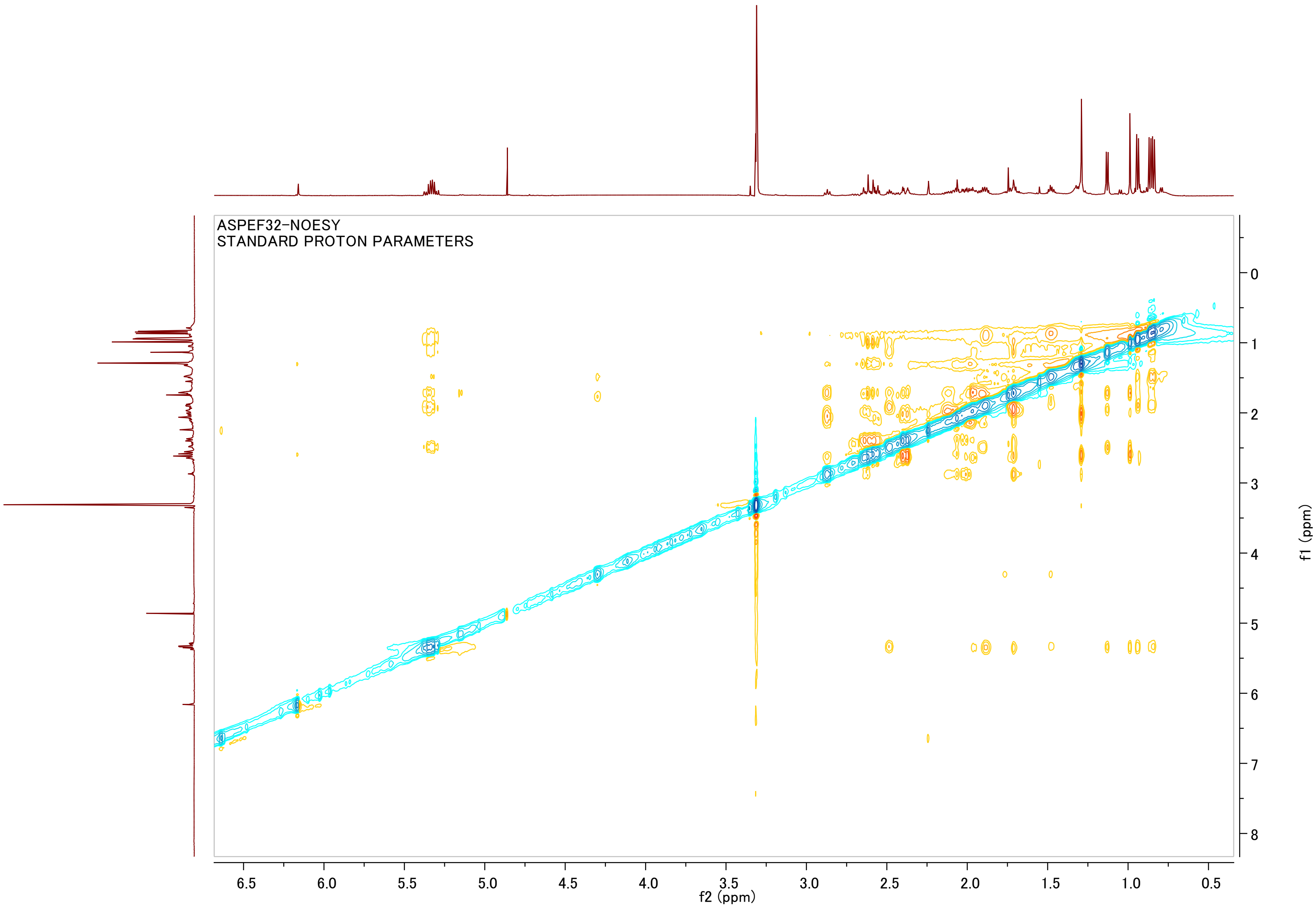

Figure S7: NOESY of versisterol.

Mass Spectrum SmartFormula Report

Analysis Info

Analysis Name	D:\Data\npchem\20180704\EBE-3-2_pos.d	Acquisition Date	7/4/2018 4:06:50 PM
Method	esi_pos_wide.m	Operator	BDAL
Sample Name	EBE-3-2	Instrument / Ser#	micrOTOF 10326
Comment			

Acquisition Parameter

Source Type	ESI	Ion Polarity	Positive	Set Nebulizer	0.4 Bar
Focus	Not active			Set Dry Heater	200 °C
Scan Begin	150 m/z	Set Capillary	4500 V	Set Dry Gas	4.0 l/min
Scan End	1500 m/z	Set End Plate Offset	-500 V	Set Divert Valve	Source

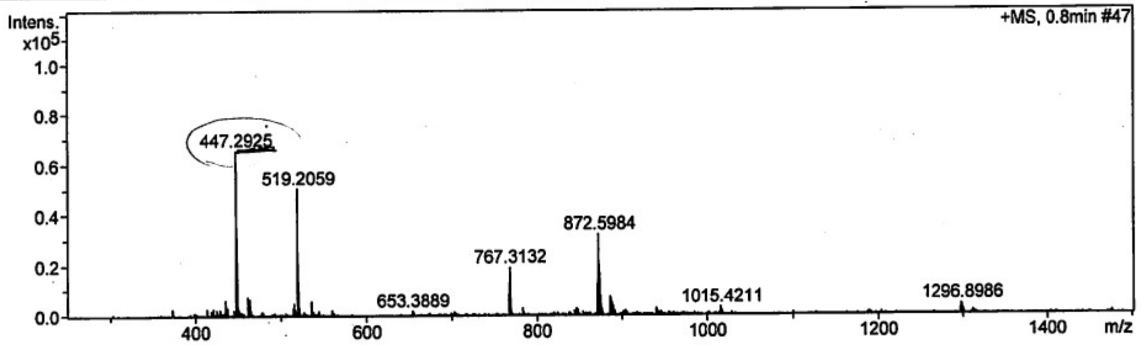

Meas. m/z	#	Formula	Score	m/z	err [mDa]	err [ppm]	mSigma	rdb	e⁻ Conf	N-Rule
447.2925	1	C 29 H 44 Na O 2	100.00	447.3234	0.0	0.0	723.2	7.5	even	ok

Figure S8: Positive HRES-ESI mass spectrum of versisterol.
